# Sarcopenia and piscines: the case for indeterminate-growing fish as unique genetic model organisms in aging and longevity research

**DOI:** 10.3389/fgene.2013.00159

**Published:** 2013-08-14

**Authors:** Jacob M. Froehlich, Zachary G. Fowler, Nicholas J. Galt, Daniel L. Smith Jr., Peggy R. Biga

**Affiliations:** ^1^Department of Biology, University of Alabama at BirminghamBirmingham, AL, USA; ^2^Department of Biological Sciences, North Dakota State UniversityFargo, ND, USA; ^3^Department of Nutritional Sciences, University of Alabama at BirminghamBirmingham, AL, USA

**Keywords:** aging, adult stem cells, senescence, indeterminate growth, sarcopenia, dynapenia, growth, Pax3/7

## Abstract

Sarcopenia and dynapenia pose significant problems for the aged, especially as life expectancy rises in developed countries. Current therapies are marginally efficacious at best, and barriers to breakthroughs in treatment may result from currently employed model organisms. Here, we argue that the use of indeterminate-growing teleost fish in skeletal muscle aging research may lead to therapeutic advancements not possible with current mammalian models. Evidence from a comparative approach utilizing the subfamily Danioninae suggests that the indeterminate growth paradigm of many teleosts arises from adult muscle stem cells with greater proliferative capacity, even in spite of smaller progenitor populations. We hypothesize that paired-box transcription factors, Pax3/7, are involved with this enhanced self-renewal and that prolonged expression of these factors may allow some fish species to escape, or at least forestall, sarcopenia/dynapenia. Future research efforts should focus on the experimental validation of these genes as key factors in indeterminate growth, both in the context of muscle stem cell proliferation and in prevention of skeletal muscle senescence.

## INTRODUCTION

As the world’s population grows past seven billion, the incidence of sarcopenia and dynapenia is expected to rise. In fact, nearly 87 million persons in the United States will be over the age of 65 by the year 2050 (Federal Interagency Forum on Aging-Related Statistics, 2008). This marked rise in elderly persons and subsequent diagnoses of sarcopenia and dynapenia pose a significant challenge to aging researchers (Clark and Manini, 2008, 2010), as the need for effective treatments (both in terms of cost and therapeutic value) for these conditions will be of utmost importance. Indeed, in 2000 alone, the United States spent over $18 billion on sarcopenia (Janssen et al., 2004), highlighting the need for increased research efforts to lower costs associated with these debilitating conditions.

Current strategies developed to combat sarcopenia and dynapenia allow for improved treatments and alternative approaches based on an increased understanding of these conditions. While clinicians have long relied on improved nutrition, specific types of exercise (whether that be simple increases in activity or targeted strength training), and management of comorbidities, there is much agreement that interventions at the level of the adult skeletal muscle stem cell, the myosatellite cell (MSC), are not only warranted, but badly needed (Machida and Narusawa, 2006; Jang et al., 2011; Thornell, 2011; Ma et al., 2012; Pallafacchina et al., 2012; Walston, 2012). Despite this deficit, no clinical trial has investigated pharmaceutical modulation of these important skeletal muscle components (Walston, 2012). Even more importantly, the poor efficacies of available therapies (Burton and Sumukadas, 2010; Brotto and Abreu, 2012; Maggio et al., 2013) are likely linked to the current model organisms used in skeletal muscle aging research.

The primary model organism for such research, the laboratory mouse, exhibits marked senescence as it increases in age. Classically, senescence (and thus sarcopenia/dynapenia) has been examined under diet or caloric restriction in several strains of mice (Selman and Withers, 2011; Yuan et al., 2011). In addition, spontaneously occurring Snell (*Pit1*^-^^/^^-^) and Ames (*Prop1*^-^^/^^-^) mice (abnormally small mice due to attenuated somatotropic axes; Brown-Borg et al., 1996; Flurkey et al., 2001), four-way and eight-way crossed mice (McClearn and McCarter, 2011), and outbred mice (Klebanov et al., 2001) have been utilized. Alternatively, mice with accelerated senescence (“SAM”) have been developed to identify genetic predispositions to aging (Chiba et al., 2009). Further, genetically engineered knockouts have proven useful in the identification of genes and/or pathways (e.g., *Surf1*, *IRS-2*, *p66Shc*, *Klotho*, *FOXO* transcription factors, *Bcl-2*) important to aging promotion or resistance (Quarrie and Riabowol, 2004). With respect to sarcopenia and dynapenia, murine models with specific mutations in the PI3K/Akt, NF-κB, myostatin/ActIIR, and autophagic pathways have proven useful for uncovering mechanisms of muscle wasting and/or gain (Romanick et al., 2013). However, no matter if the mouse is of inbred, outbred, crossed, or transgenic origin, it is still a *mouse*, and although increases in lifespan have been achieved, these extensions are still accompanied by marked senescence *at some point*, accompanied with marked sarcopenia/dynapenia and not without invasive genetic interventions. Put another way, no mouse model is useful in studying an *escape* from senescence, limiting major breakthroughs in the field of aging research.

While limited studies in mammals appear to indicate that smaller body size within a species may be correlated with increased lifespan (Bartke, 2012), vertebrates across a spectrum of sizes, from olms (Voituron et al., 2011), rockfish (Cailliet et al., 2001; Munk, 2001), sturgeons (Sulak and Randall, 2002), orange roughy (Fenton et al., 1991), warty oreo (Stewart et al., 1995), tortoises (Gibbons, 1987), and bowhead whales (George et al., 1999), can exceed human life spans, living as long as >200 years in some species. This has led to the “negligible senescence” hypothesis, first proposed by Finch (1992). Under this paradigm, animals with negligible senescence exhibit little to no decline in reproductive function, physiological status, or increased mortality with age (Finch, 1990). As seen in the list above, many of these animals are teleost fish. However, the many confounding variables between rodents and teleosts (e.g., terrestrial vs. aquatic, actinopterygian vs. tetrapod) make direct juxtaposition of these species difficult at best.

Of all the biological differences between rodents (and mammals in general) and teleost fish, the most important may be the contrasting growth potentials of these two groups of animals. Laboratory rodents, like nearly all mammals and including humans, reach a definitive size following puberty; that is, they exhibit a characteristic growth plateau (Lui and Baron, 2011) and are considered “determinate” growers, as defined by Lincoln et al. (1982). Under this paradigm, genetics govern growth, with some significant environmental intervention (Sebens, 1987). However, piscine biologists have long known that many fish do not appear to possess such a strict growth plateau (Sebens, 1987), as they continue to grow throughout their lives, albeit at a slower rate. This type of growth, termed “indeterminate,” is very common among many fish species (and invertebrates, although they are outside the scope of this article) while it is believed to be absent in most terrestrial vertebrates. In contrast to mammals, the indeterminate growth observed in most fishes is highly influenced by environmental factors such as temperature, competition, and food availability (Sebens, 1987). This paradigm is one in which age is highly predictive of body size (Lincoln et al., 1982), a contrast with determinate growth.

With respect to skeletal muscle, a tissue with high metabolic activity constituting a large proportion of the mass of vertebrates, the differences between terrestrial mammals and aquatic piscines continue. Elegantly demonstrated by Rowe and Goldspink (1969), eutherian skeletal muscle (modeled by *Mus musculus*) is augmented primarily by hypertrophy of existing myofibers in the postnatal period. Conversely, Stickland (1983) demonstrated that adult rainbow trout (*Oncorhynchus mykiss*) skeletal muscle of both fiber types can undergo myofiber hypertrophy *and* hyperplasia, recruiting nascent myofibers well into the postlarval period without injury or trauma. Outside the salmonid clade, other fishes, namely cyprinids such as the common carp (*Cyprinus carpio*; Alfei et al., 1994) and giant danio (*Devario aequipinnatus*; Biga and Goetz, 2006), and acipenseriform fish like sturgeon (Daczewska and Saczko, 2005) appear to grow in a similar manner to the rainbow trout. This represents an increase in organismal size by a magnitude of 10^5^–10^8^ between the larval and adult periods of their life histories, the bulk of which is skeletal muscle. How do fish accomplish such feats of growth? Even more interestingly, how do fishes continue to accumulate new muscle fibers even into old age? The answers to such questions are critical for attenuating the effects of sarcopenia and dynapenia in the increasingly large aged population. However, while studies of teleost fish may be attractive to aging researchers of diverse disciplines, none of the fishes named above are easily kept in the laboratory, readily available, or easily propagated, as is the case with many of the other vertebrates known to possess extreme lifespans. Thus, a laboratory model of slow to negligible aging and indeterminate growth is needed.

## THE CASE FOR CELL NUMBER: IS IT A MATTER OF MAGNITUDE?

The growth potential of teleost fish leads to the obvious prediction that these vertebrates possess resident adult stem cells in their skeletal muscle with greater potency and an increased ability to replenish the pool of progenitor cells than do terrestrial vertebrates like mammals. However, direct juxtaposition between fish and mammals are difficult due to the inherent differences in anatomy and physiology, especially in skeletal muscle tissue (i.e., fish “red” and “white” myofibers are separate while mammalian slow-oxidative and fast-glycolytic fibers are intermixed). Fast-glycolytic, type II myofibers constitute the bulk of the teleost myotome, with a small band of type I fibers running parallel to the lateral line (Hollway and Currie, 2005; Rescan, 2005). These anatomical features of fish make them even more attractive for sarcopenia/dynapenia research, as sarcopenia is often accompanied by a conversion of type II fibers to type I fibers (von Haehling et al., 2010). The observation that many indeterminate-growing fish can evade sarcopenia all the while maintaining such a large proportion of type II fibers supports their use in such research, especially since MSC populations associated with type II fibers decline in sarcopenia (Verdijk et al., 2007).

**FIGURE 1:**
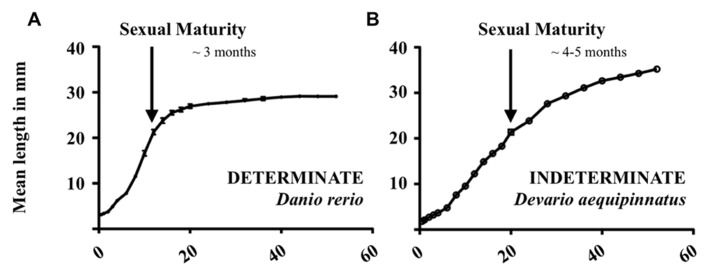
**Growth curves of **(A)** zebrafish (*Danio rerio*) and **(B)** giant danio (*Devario aequipinnatus*) from hatch to 60 weeks of life.** Mean length in millimeter; *n* = 10 per time point.

Conveniently, the Danioninae clade (in addition to a few other groups of fish, namely Poeciliidae, and the medaka, *Oryzias latipes*; Veggetti et al., 1993; Hatakeyama et al., 2008) includes species of both growth paradigms, namely the zebrafish (*Danio rerio*) and giant danio (*Devario aequipinnatus*; Biga and Goetz, 2006; Tang et al., 2010). Within the first 60 weeks of life, the growth trajectories of zebrafish (**Figure 1A**) and giant danio (**Figure 1B**) are drastically different, with an obvious growth plateau being reached in the former. The larger, indeterminate giant danio, however, continues to grow without such an asymptote. Under strong growth promotion (i.e., administration of growth hormone, GH), these differences hold true, with the determinate-like zebrafish maintaining a growth plateau while the indeterminate giant danio is able to modulate its growth trajectory (Biga and Goetz, 2006).

**FIGURE 2:**
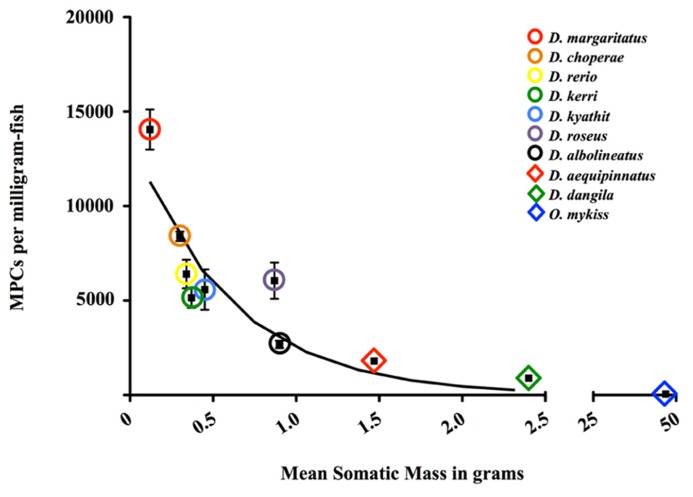
**Total number of myogenic precursor cells (MPCs) isolated from select Danioninae species (Cypriniformes: Cyprinidae: Danioninae) and one salmonid (Salmoniformes: Salmonidae: Salmoninae; *Oncorhynchus mykiss*) per milligram-fish under normal growth conditions is related to the overall mean body mass of the fish (*y* = -0.7065 × +3.498; *R*^**2**^ = 0.8205; log–log regression).** Circles (◯) and rhombuses (♦) represent determinate and indeterminate fish, respectively.

Because upward of 60% of the mass of a teleost fish is constituted by skeletal muscle, we hypothesized that myogenic precursor cells (MPCs), the adult stem cells of skeletal muscle, play an integral role in the lifetime growth potential (or lack thereof) of danionin fishes and likely all teleost fish. Using a well-established protocol for the isolation of such cells, we determined that each “determinate-like” zebrafish, appears to hold approximately 2.2 million MPCs (2.208 × 10^6^ ± 6.281 × 10^3^; *n* = 6; see **Figure 2**). The same appears to be true for the indeterminate giant danio (**Figure 2**), with each fish possessing 2.2 million MPCs (2.242 × 10^6^ ± 3.458 × 10^3^; *n* = 6). However, giant danios’ are *much* larger than their true *Danio* cousins (on the order of three times). Correcting for mass, 6.404 × 10^3^ MPCs/mg-muscle reside in the fast-glycolytic epaxial myotome of each zebrafish while a mere 1.798 × 10^3^/mg-muscle can be found in the larger giant danio. With respect to other members of the Danioninae subfamily, the trend is the same. The smallest species examined, the celestial pearl danio (*Danio margaritatus*), yields around 1.2 million MPCs per fish (1.225 × 10^6^ ± 2.571 × 10^3^; *n* = 6) or approximately 11000 MPCs/mg of skeletal muscle (1.100 × 10^4^ ± 2.268 × 10^3^; *n* = 6; see **Figure 2**). While the celestial pearl danio is only one-tenth the size of its much larger cousin (the giant danio), its epaxial musculature holds more than six times as many MPCs. Interestingly, intermediate-sized danionins’ (e.g., rosy danio, *Danio roseus*, and pearl danio, *Danio albolineatus*) possess an intermediate number of MPCs (**Figure 2**). While giant danios are not true *Danio*, similar cell populations are found in the similarly sized mustached danio (*Danio dangila*; **Figure 2**). Strikingly, the rainbow trout (*Oncorhynchus mykiss*) possesses the fewest MPCs, with 5.175 × 10^1^ ± 2.566 × 10^1^ MPCs/mg of skeletal muscle. Thus, it appears that larger fish have remarkably *fewer* MPCs and cell population size cannot account for the opposing growth paradigms. How then do fish accomplish lifetime growth in their musculature with such few MPCs? We hypothesize that *enhanced self-renewal of adult stem cells (MPCs in skeletal muscle) leads to the indeterminate growth paradigm, with subsequent differential fusion later in myogenesis in the case of skeletal muscle****. ***This phenotype is likely linked to differential gene expression or pathway signaling, holding promise for aging research.

Where do murine MPCs (MSCs) fall on this spectrum? Bentzinger et al. (2012) estimated that a 20–30 g mouse (C57Bl6/J background) possesses ≈4 × 10^6^ MSCs or 1.20 × 10^2^ MSCs/mg of mouse. While this number may fall between the indeterminate danios (giant and mustached) and the indeterminate rainbow trout (see **Figure 2**), direct comparison is highly confounded. Mouse MSCs are identified by Pax7 expression (Bentzinger et al., 2012), with large differences in number of MSCs between fast- and slow-twitch fibers – on the order of two to three times (Collins et al., 2005; Shefer et al., 2006). As discussed above, fiber types in teleosts are separate, with the vast majority of the musculature being constituted by fast-twitch fibers (the source of our MPCs). In fact, total fiber number of both types remains unchanged from birth to 24 weeks in mice (well beyond sexual maturity), while fast-glycolytic fiber number in rainbow trout grows by sixfold over just 65 cm of growth (Rowe and Goldspink, 1969; Stickland, 1983). Additionally, recent data from our laboratory indicates that Pax7 expression, at least in the indeterminate giant danio, appear to possess very few Pax7^+^ MPCs (Froehlich et al., 2013), further confounding the comparison.

## SELF-RENEWAL: GROWING THE POOL WITH MORE PROGENY

**FIGURE 3:**
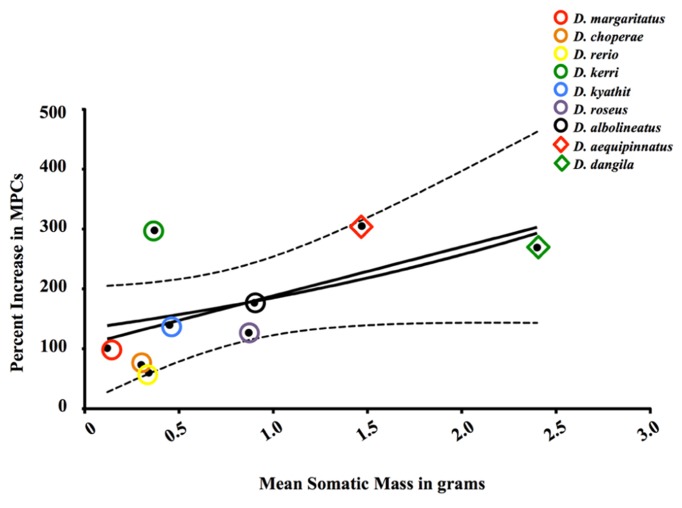
**Percent-change in population of MPCs isolated immediately *ex vivo* from select Danioninae species (Cypriniformes: Cyprinidae: Danioninae) following four injections of 120 μg ovine GH per gram fish over 2 weeks in relation to mean body mass in grams [*y* = 133.5 × 10^(0.3279 ×)^; *R*^**2**^ = 0.1023; linear regression].** Circles (◯) and rhombuses (♦) represent determinate and indeterminate fish, respectively.

Higgs et al. (1975) demonstrated that GH administration could stimulate somatic growth in Coho salmon (*Oncorhynchus kisutch*). This phenomenon is unlike that seen in mammals, as GH can only stimulate muscle hypertrophy to a maximal size specifically and does not stimulate overall body growth generally in adult mammals (Shea et al., 1987). For lifetime growth to occur in fishes, muscle hyperplasia and recruitment of nascent myofibers must occur. As noted by Johnston et al. (2011), hypertrophy of existing myofibers is limited to the critical diameter of those fibers. Thus, new fibers must be generated *de novo* for indeterminate growth to occur and it has been hypothesized that MPCs, like their mammalian kin known as MSCs, generate the necessary cellular progeny to form such fibers through myoblast-to-myoblast fusion. However, the demonstration that small fish like zebrafish and even guppies (Veggetti et al., 1993; Biga and Goetz, 2006) do not undergo *appreciable* muscle hyperplasia as adults suggests that MPCs from species with opposing growth paradigms differ in their ability to proliferate and generate additional MPCs (i.e., “determinate-like”). As shown by Biga and Meyer (2009), determinate-like fish respond differently to exogenous GH administration (a potent stimulator of growth) at the molecular level [e.g., insulin and insulin-like growth factor-I (IGF-I) receptors and the chalone myostatin] than do their indeterminate relatives. From this data, we predict that similar differences are present at the cellular level. Indeed, the MPCs of the minute *Danio margaritatus*, the smallest of the danionins examined (*n* = 6 per species; **Figure 3**), appear to be less sensitive to GH, as this cellular population is augmented by approximately 101% following GH administration. Similarly, the intermediate-sized fish such as the rosy danio (*Danio roseus*) and the pearl danio (*Danio albolineatus*) are able to increase MPC numbers by 127 and 177%, respectively. Most interestingly, the MPCs of the indeterminate giant danio undergo the most cellular proliferation (fivefold greater increase in giant danio MPCs compared to zebrafish and 305% of control levels; **Figure 3**). These data, in tandem with those collected under steady-state conditions, highly suggest that phenotypes, not numbers, of teleost MPCs afford lifelong skeletal muscle growth, and thereby somatic growth, in these fishes.

As under steady-state levels, comparisons of growth and MPC/MSC expansions between fishes and mammals are difficult at best. However, administration of GH to determinate-growing mammals does not produce appreciable muscle hyperplasia, as predicted by their growth paradigm (Dudley and Portanova, 1987; Sharma et al., 1996; Schuenke et al., 2008). While *in vivo* data of mammalian MSC behavior in response to GH are lacking, GH does *not *appear to promote MPC/myoblast proliferation but does enhance fusion, at least primary *in vitro *culture (Sotiropoulos et al., 2006). This contrasts with a previous report of *in vitro *GH-stimulated proliferation in an indeterminate-growing salmonid, the Atlantic salmon (*Salmo salar*), under similar conditions (Levesque et al., 2008). Despite this alluring lead, a more appropriate comparative system, such as the one described herein, is needed to determine whether growth paradigm directly influences MPC/MSC proliferative and fusion behavior (i.e., low vs. high; myoblast-to-myofiber fusion vs. myoblast-to-myoblast fusion).

## Pax GENES: POWERFUL POTENTIATORS OF PROLIFERATION?

How can these large differences in MPC proliferation exist in such closely related fishes like the danionins discussed above? We hypothesize that differential gene regulation has evolved in this clade (and possibly in others containing small, determinate-like fishes like guppies) and likely involves a suite of traditional *cis*- and *trans*-modulations and epigenetic modifications of protein expression. In light of these findings, several targets are ideal for investigation. Transcription factors of the paired-box (Pax) family have been definitively implicated in skeletal myogenesis (Williams and Ordahl, 1994; Seale et al., 2000; Lepper et al., 2009). Specifically, Pax3 and Pax7 are differentially expressed during developmental, growth, and reparative stages of myogenesis (Buckingham and Relaix, 2007; Lagha et al., 2008). Interestingly, Pax3 appears to be most important during the embryonic (primary) stages of myogenesis and not during adulthood (except in the diaphragm, muscle spindles and isolated body wall and hindlimb muscles, where it does appear to be expressed during postnatal life). Conversely, Pax7 plays a role during secondary (fetal) and neonatal specification of MPCs, although it appears to be dispensable outside of the neonatal period (Lepper et al., 2009). In mammals, muscle fiber hypertrophy and hyperplasia occur only during primary and secondary myogenesis (Biressi et al., 2007).

The ontogeny of development and growth in fish, however, is much different. While hypertrophy of myofibers occurs throughout piscine life histories, fish pass through two stages of muscle hyperplasia during development and growth: stratified and mosaic hyperplasia. In stratified hyperplasia, a distinct region of hyperplastic nascent fibers develops in the epaxial myotome (Johnston and McLay, 1997). Mosaic hyperplasia occurs later in life and involves the recruitment of nascent myofibers throughout the myotome (Weatherley and Gill, 1987). *Prima fascia*, it appears that secondary myogenesis in mammals and mosaic hyperplasia in fish are analogous processes. The demonstrated role of Pax3 during murine embryonic development (but its widespread absence in MPCs of juvenile and adult origins) solicits questions about the role of Pax3 and Pax7 during piscine development and postlarval myogenesis. Pax7, the perennial marker of MPCs in mammals, plays a key role in the self-renewal of these cells (Rocheteau et al., 2012). While Pax genes appear to be well conserved in zebrafish (Seo et al., 1998), little is known about the roles of these genes during postlarval growth. Only in sea lamprey (*Lethenteron japonicum*; Kusakabe and Kuratani, 2007) and zebrafish (Hammond et al., 2007) has Pax3 expression been examined (albeit during embryonic and larval development and largely in neural crest cells). It must be noted, though, that Pax3 and Pax7 are restricted to a single orthologous gene in lamprey. Additionally, we are aware of only two studies in which Pax3 has been examined in the context of skeletal muscle growth and repair in fish. In larval zebrafish, Seger et al. (2011) found Pax3^+^ MPCs remained unchanged during reparative myogenesis while Pax7^+^ MPCs appear to be most responsible for injury repair. However, Pax3 has been suggested to play a key role in fiber maintenance (Kirkpatrick et al., 2010), a finding that may be more relevant to lifelong growth than injury. *In vitro*, we recently demonstrated that Pax3 appears to be important in the proliferation of myoblasts isolated from giant danio (Froehlich et al., 2013), lending further support to our hypothesis. With respect to Pax7 in fish, this factor appears to be important not only in early myogenesis, but even in later stages. In the indeterminate-growing Atlantic salmon (*S. salar*), *Pax7* transcripts appear to be upregulated during *in vitro* myogenesis (even in late-stage myoblasts), a finding that appears to contrast with that seen in mammals (Bower and Johnston, 2010). Indeed, recent microarray data from rainbow trout indicate that Pax3 and Pax7 are in fact upregulated during hyperplasia (Rescan et al., 2013). Thus, we hypothesize that indeterminate fish species like salmonids and giant danio are able to accomplish continued growth well into adulthood through self-renewal of MPCs via a Pax3/7-dependent mechanism.

## SARCOPENIA IN FISH: DOES IT EXIST?

The lifelong growth of many teleosts begs this question: does the existence of an asymptotic growth plateau lead to senescence? Studies utilizing the model teleost, the zebrafish, have provided some insight into this question. While zebrafish appear to age much slower than do rodents, signs of senescence are nonetheless present. It has been well documented that zebrafish display pathological curvatures of the spine, muscle wasting akin to sarcopenia, and failure of maintain position in the water column (Gerhard et al., 2002) as they age. In our laboratory, we have witnessed similar phenotypes in aged zebrafish strains, including AB, WIK, TU, and Tupfel long fin animals. In commonly used “outbred” (pet store) strains of zebrafish, the median age of longevity has been determined to be 3.5 years, with the oldest adults living until 5.5 years (Gerhard et al., 2002). Inbred strains (such as those listed above) display decreased life spans, with a median of 3 years (Gerhard et al., 2002), and wild zebrafish survive for even shorter periods of time (Spence et al., 2008). Data describing the lifespans of other danionins are scare to non-existent. Personal communications with aquarists suggests that most danionins live until the ages of 2–5, with some of the larger species living until ages 5–7. FishBase, an online repository of ichthyological data (Froese and Pauly, 2013), predicts similar lifespans for most danionins (see **Table 1**). Importantly, age does not appear to fetter growth trajectories in the indeterminate danionins (Biga and Goetz, 2006), further implicating growth paradigm in the development of sarcopenia.

**Table 1:** Approximate lifespans of select danionins.

Species	Common name	Approximate lifespan (year)	Reference
Small danionins	Celestial pearl danio, glowlight danio, zebrafish, blue danio, spotted danio	1.0–3.5	FishBase (Gerhard et al., 2002; Spence et al., 2008)
Medium danionins	Rosy danio, pearl danio	1.8–3.1	FishBase
Large danionins	Mustached danio, giant danio	4.0–6.0	FishBase (P. W. Cottle, personal communication)

With respect to skeletal muscle, fibrosis, senescence-associated β-galactosidase activity (Kishi et al., 2003), and protein oxidation (Dimri et al., 1995) have been documented in zebrafish. However, its kin, the giant danio, displays an apparent resistance to aging. In our laboratory, we have never observed giant danios with pronounced characteristics of aging, even in fish aged 4–5 years, an age where zebrafish do appear senescent. In larger indeterminate fish, like rockfish and warty oreo, lifespans of ~200 years are possible, even without loss of reproductive output (Stewart et al., 1995; Cailliet et al., 2001). Whilst some larger fish (i.e., several species of *Oncorhynchus* salmonids) do exhibit drastic senescence and death following spawning, this phenotype is likely linked to the semelparity of these species and not indeterminate growth (Crespi and Teo, 2002). In general, however, growth indeterminacy holds promise for skeletal muscle aging research, and research using fish species with indeterminate growth should be a top priority for future investigations, namely the description of and experimental perturbation of mechanisms of continued and exhaustion-resistant self-renewal of MPC/MSC populations and the resulting effects on growth paradigm.

## CONCLUSION

**FIGURE 4:**
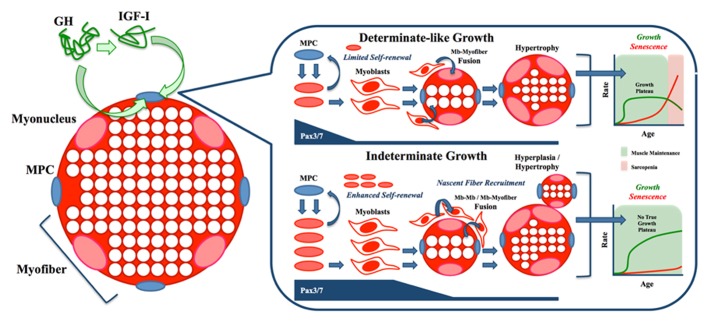
**Working model of opposing growth paradigms.** Stimulation of somatic growth by growth hormone (GH) and insulin-like growth factor-I (IGF-I) leads to prolonged Pax3/7 expression, resulting in enhanced myogenic precursor cell (MPC) proliferation, expansion of myoblast (Mb) progeny and increased self-renewal of the stem cell niche. Downstream of these effects, differential fusion potential results largely in hypertrophy of existing fibers in determinate organisms (i.e., mammals) and nascent fiber recruitment in indeterminate organisms (i.e., many fish). At the most ultimate level, a growth plateau (or asymptote) may predispose animals to sarcopenia, while the presence of lifelong, indeterminate growth may preclude senescence, including sarcopenia/dynapenia.

The presence of a growth plateau in zebrafish and many mammals appears to be consistent with the marked senescence, particularly in skeletal muscle, seen in these species. In contrast, the vast majority of teleosts do not seem to undergo such a process, a phenotype that may be linked to their lifelong, non-asymptotic growth generally and their ability to recruit nascent myofibers proximately. The central role of Pax transcription factors in myogenesis and their likely function in the self-renewal and proliferation of myogenic progenitors of fish (among other vertebrates) makes them prime candidates for the study of sarcopenia evasion in indeterminate teleosts. As **Figure 4** illustrates, we hypothesize that the continued self-renewal of MPCs needed for lifelong growth (shown in green) leads to minimal aging (shown in red), a paradigm not seen in fishes with a growth plateau, where the inability to sustain growth results in eventual muscle wasting (sarcopenia). However, this hypothesis can only be tested using an unconfounded model system, one exemplified by the danionin fishes.

While the indeterminate rainbow trout has been utilized by fish biologists for decades, it has not received the attention of aging researchers, largely due to its size, somewhat difficult husbandry, and lack of genomic resources. Even as the rainbow trout genome sequence nears completion, we are unaware of any directly comparable species within the salmonid family. Capitalizing on the model organism status of the zebrafish, large, indeterminately growing danionins such as the giant and mustached danio hold much promise improving the efficacy of current sarcopenia/dynapenia treatments and advancing the discovery of new and innovative drug targets to combat these pervasive conditions, provided that the necessary husbandry and breeding protocols, life history, and senescence-related data are available.

## Conflict of Interest Statement

The authors declare that the research was conducted in the absence of any commercial or financial relationships that could be construed as a potential conflict of interest.
